# Metabolomic Signatures of Autoimmune Hepatitis in the Development of Cirrhosis

**DOI:** 10.3389/fmed.2021.644376

**Published:** 2021-03-12

**Authors:** Shan-shan Li, Ming Niu, Jing Jing, Ying Huang, Zi-teng Zhang, Shuai-shuai Chen, Ge-zi Shi, Xian He, Hai-zhu Zhang, Xiao-he Xiao, Zheng-sheng Zou, Yue-cheng Yu, Jia-bo Wang

**Affiliations:** ^1^School of Pharmacy and Chemistry, Dali University, Dali, China; ^2^Department of Liver Diseases, Fifth Medical Center of Chinese PLA General Hospital, Beijing, China; ^3^Department of Poisoning Treatment, Fifth Medical Center of Chinese PLA General Hospital, Beijing, China; ^4^Liver Diseases Center of General Hospital of PLA Eastern Theater Command, Bayi Hospital Affiliated to Nanjing University of Chinese Medicine, Nanjing, China; ^5^School of Traditional Chinese Medicine, Capital Medical University, Beijing, China

**Keywords:** liver cirrhosis, autoimmune hepatitis, metabolomics, metabolic pathway, biomarkers

## Abstract

**Objectives:** Autoimmune hepatitis (AIH) can progress into severe outcomes, i.e., decompensated cirrhosis, from remarkable and persistent inflammation in the liver. Considering the energy-expending nature of inflammation, we tried to define the metabolomics signatures of AIH to uncover the underlying mechanisms of cirrhosis development and its metabolic biomarkers.

**Methods:** Untargeted metabolomics analysis was performed on sera samples from 79 AIH patients at the stages (phenotypes) of non-cirrhosis (*n* = 27), compensated cirrhosis (*n* = 22), and decompensated cirrhosis (*n* = 30). Pattern recognition was used to find unique metabolite fingerprints of cirrhosis with or without decompensation.

**Results:** Out of the 294 annotated metabolites identified, 2 metabolic fingerprints were found associated with the development of cirrhosis (independent of the decompensated state, 42 metabolites) and the evolution of decompensated cirrhosis (out of 47 metabolites), respectively. The cirrhosis-associated fingerprints (eigenmetabolite) showed better capability to differentiate cirrhosis from non-cirrhosis patients than the aminotransferase-to-platelet ratio index. From the metabolic fingerprints, we found two pairs of metabolites (Mesobilirubinogen/6-Hydroxynicotinic acid and LysoPA(8:0/0:0)/7alpha-Hydroxycholesterol) calculated as ratio of intensities, which revealed robust abilities to identify cirrhosis or predict decompensated patients, respectively. These phenotype-related fingerprint metabolites featured fundamental energy supply disturbance along with the development of AIH cirrhosis and progression to decompensation, which was characterized as increased lipolysis, enhanced proteolysis, and increased glycolysis.

**Conclusions:** Remodeling of metabolism to meet the liver inflammation-related energy supply is one of the key signatures of AIH in the development of cirrhosis and decompensation. Therefore, drug regulation metabolism has great potential in the treatment of AIH.

## Introduction

Autoimmune hepatitis (AIH) is an inflammatory disease of the liver mediated by an abnormal autoimmune response (1). The pathogenesis of AIH is not fully understood. AIH patients may have long-term asymptomatic tissue inflammation, but they are often not diagnosed, thus losing treatment opportunities. When clinical symptoms appear, the disease may have progressed to a more severe stage, and may even further progress to cirrhosis, decompensation of liver function and eventually liver failure (2, 3). AIH cirrhosis has no specific clinical manifestations in the compensated stage; When the disease progresses to the decompensated stage, liver dysfunction and portal hypertension will appear. Decompensated patients may also experience symptoms such as gastrointestinal bleeding, liver ascites, and coma. Liver transplantation is an effective treatment for decompensated patients (4), but AIH may recur after transplantation. Although AIH is a disease mediated by autoimmune abnormalities and autoantibodies are important for the diagnosis of AIH, serum autoantibodies do not reflect the severity of the disease in AIH patients (5). Looking for biomarkers that can predict the progression of liver cirrhosis and malignancy in AIH patients is of immense significance in the clinical prognosis and understanding of the underlying mechanism of disease progression.

AIH is mainly characterized by liver inflammation, which is essentially a chronic and unresolved liver inflammatory damage (6). Inflammation is an energy-expensive biological process that requires a large amount of energy and intermediary metabolites for the synthesis of inflammatory factors and immune response (7). Continuous hepatic inflammation inevitably causes significant metabolic changes and induces a wide range of catabolic dysfunction, which may be involved in the progression of the disease. The liver is the main metabolic organ, responsible for most of the body's metabolic processes such as synthesis, decomposition, and transformation (8), thus, it is very important to study AIH from the perspective of metabolism.

Metabolomics is a phenotypic method for studying metabolites, small molecule substrates, and intermediary metabolites, which directly reflects potential biochemical activity and state of cells/tissues (9). Furthermore, it is a powerful method to explore specific biomarkers of liver diseases (10). In recent years, non-targeted metabolomics has been applied to the study of AIH-related biomarkers (11–13), but there is no research related to disease progression of AIH.

Here, we report the findings of untargeted and whole spectrum metabolomic study in sera from 79 AIH patients and compare the metabolic markers of liver cirrhosis and decompensation. The aim of this study is to characterize the sera metabolite fingerprints specific to patients with compensated or decompensated AIH cirrhosis, and to identify potential prognostic biomarkers of AIH.

## Materials and Methods

### Study Design

We explored the sera samples of hospitalized AIH patients in the Biobank of Fifth Medical Center of Chinese PLA General Hospital from October 31st, 2015 to May 19th, 2017. Patients diagnosed with AIH and where adequate clinical information was available, were selected for the study. This research scheme was approved by the Ethics Committee of the hospital based on the ethical principles of the Declaration of Helsinki, and written informed consent was obtained from all patients. Their sera had to be deposited within 4 days after diagnosis. Patients with competitive etiologies were excluded. There are 89 AIH patients initially screened, and 10 of them were excluded due to competitive etiologies. Among those 10 excluded patients, 8 had concurrent drug-induced liver injury and 2 had alcoholic liver disease. Finally, 79 patients were enrolled in the study. The enrolled samples were divided into three groups: non-cirrhosis (NC) (*n* = 27), compensated cirrhosis (CC) (*n* = 22), and decompensated cirrhosis (DC) (*n* = 30) groups, according to the international clinical guidelines (14). Based on untargeted metabolomics, we acquired numerous metabolites (metabolome profile) with significant difference (*P* < 0.05 and FC >1.5 or <0.67) by comparing CC and DC with NC and marked them as (CC+DC)/NC. Metabolites identified in metabolome profiles were considered as ones with cirrhosis-associated metabolome features. Similarly, by comparing CC or DC with NC, they were marked as CC/NC and DC/NC, respectively. The excluded part of DC/NC can be considered as decompensation-associated metabolome features. Also, hierarchical clustering was used to screen out the metabolic fingerprints, which consisted of a cluster of metabolites with high area under curve (AUC) of the receiver operating characteristic (ROC) analysis and *P*-values, in differentiating each group (phenotype). Furthermore, the phenotype-associated fingerprint metabolites were projected to one eigenmetabolite by reducing the dimension, which is defined as the first principal component in unsupervised principal component analysis (PCA) (15). The eigenmetabolite was further investigated to discover relationships with demographics, biochemistry, complications, and clinical evaluation models. Finally, metabolic pathways of phenotype-associated fingerprint metabolites were analyzed to interpret the underlying mechanisms of the metabolic drivers in the evolution of AIH.

### Samples Preparation and Chromatographic Conditions

The biobanked serum were processed according to the literature (16). Quality control samples (QC) were prepared by mixing 10 μL from each sample to be analyzed. Chromatographic column: ZORBOX RRHD C18 analytical column (2.1 mm i.d. × 100 mm, 1.8 μm i.d., Agilent Technologies, USA). Column temperature: 30°C. Sample temperature: 4°C. The mobile phase was composed of solvent A: Water with 0.1% formic acid in positive mode of Q-TOF, pure water in negative mode, mobile phase B: Acetonitrile. Flow rate: 0.30 mL/min; Sample injection size: 4 μL. Chromatographic gradient elution conditions: 0–1 min, 100% (A); 1–9 min, 100%-60% (A); 9–19 min, 60–10% (A); 19–21 min, 10–0% (A); 21–25 min, 100% (B).

### Mass Spectrum Condition

An Agilent 6550 Q-TOF LC/MS with an electrospray ionization source (ESI) in positive and negative ion modes was used. Non-targeted primary mass spectrometric detection conditions: The mass range of m/z 50–1,200; gas temperature of 225°C in both positive and negative ionization modes; airflow of 13 l/min; the nebulizer of 20 pisg (negative) and 20 pisg (positive); sheath gas temperature of 275°C and sheath gas flow of 12 l/min; electrospray capillary voltage of 3,500 V in negative ionization mode and 4,000 V in positive ionization mode; and nozzle voltage of 2,000 V in the positive and negative ion modes. The acquisition mode was ESI Continuum mode and 14,104 variables were captured in positive ion mode as well as 13,357 variables were captured in negative ion mode.

### Statistical Analysis

All variables were normalized in MetaboAnalyst 4.0 (https://www.metaboan-alyst.ca) after being preprocessed in Masshunter Profinder software. Summarized results of the patients' clinical biochemical characteristics for continuous variable are expressed as median (upper quartile, lower quartile), and categorical variables are expressed as numbers and percentage. Comparisons between groups were made with Kruskal-Wallis H test. All statistical analysis was performed using SPSS 21.0 software. The significance level for all statistical tests was set at 0.05. PCA and orthogonal partial least squares discrimination analysis (OPLS-DA) were performed in SIMCA -P 14.1 software. The *P*-values, fold change (FC) and AUC of ROC were obtained from the MetaboAnalyst website. We narrowed down the original variables to fingerprint by *P*-value, FC, and AUC.

## Results

### Baseline Demographic and Clinical Characteristics of the Study Cohort

The baseline demography and clinical characteristics among NC, CC, and DC groups are described in Table 1. There was no difference in the mean ages among the three groups. Except for 6 male patients, female patients accounted for an overwhelming proportion of AIH patients (*n* = 73, 92.41%). Between NC and CC groups, the levels of TBA, PT, and INR in CC were much higher than those in NC, and the levels of CHE, ALB, PLT, and WBC in CC were significantly lower than those in NC. Significantly lower levels of ALB and CHE were found in DC groups as compared to the CC group. With respect to complications, 86.67% of patients in the DC group had ascites. Besides, there was no hepatic encephalopathy in AIH patients. Aminotransferase-to-platelet ratio index (APRI) score showed an increasing trend along with the development of cirrhosis from NC to CC and DC.

**Table 1:** Clinical information comparison between liver disease patients with NC, CC, and DC.

	**Non-cirrhosis (*n* = 27)**	**Compensated cirrhosis (*n* = 22)**	**Decompensated cirrhosis (*n* = 30)**	***P^***a***^***	***P^***b***^***
Age/year	51(42,56)	54(50,63)	57.5(51.0,67.0)	0.224	0.274
Female, *n* (%)	27(100.0)	18(81.8)	28(93.3)	–	–
ALT/U·L^−1^	30.0(12.0,96.0)	35.5(20.3,145.8)	32.0(22.0,76.8)	0.26	0.663
AST/U·L^−1^	59.0(26.0,121.0)	75.0(30.8,171.5)	49.5(36.8,117.8)	0.148	0.788
AST/ALT	1.4(1.1,2.2)	1.6(0.9,2.4)	1.8(1.3,2.7)	0.702	0.404
ALP/U·L^−1^	105.0(79.0,184.0)	135.0(114.8,190.8)	118.5(90.3,224.0)	0.067	0.437
GGT/U·L^−1^	67.0(15.0,197.0)	94.0(48.3,164.3)	49.5(26.3,181.5)	0.501	0.47
TBil /mg·dl^−1^	14.2(10.2,22.4)	22.3(13.5,61.4)	26.6(11.8,83.5)	0.03	0.624
DBil/μmo·L^−1^	5.7(3.3,12.0)	11.6(5.3,47.1)	14.5(5.4,66.5)	0.027	0.657
IgA/g·L^−1^	2.6(1.9,3.5)	2.9(1. 9,4.6)	3.7(2.4,6.2)	0.488	0.154
IgG/g·L^−1^	15.0(12.1,21.3)	21.6(15.5,24.0)	20.4(16.2,25.8)	0.062	0.97
IgM/g·L^−1^	1.2(0.8,1.7)	1.7(0.9,2.9)	1.7(1.1,2.3)	0.093	0.795
CHE/U·L^−1^	5241.0(4877.0,7125.0)	4192.0(2938.3,6100.5)	2883.0(1984.8,4037.3)	0.011	0.005
INR/IU	1.0(0.9,1.0)	1.1(1.0,1.2)	1.1(1.0,1.3)	0.005	0.194
TC/mmol·L^−1^	4.2(3.7,5.0)	3.8(2.8,4.7)	3.3(2.7,4.0)	0.151	0.243
TG/mmol·L-1	1.1(0.9,1.5)	1.3(0.8,1.7)	1.0(0.8,1.6)	0.96	0.535
ALB/g·L-1	36.0(34.0,40.0)	33.0(29.5,37.0)	29.5(25.8,33.3)	0.025	0.05
WBC/mm^3^	5140.0(4340.0,6800.0)	3825.0(3155.0,4777.5)	3850.0(2852.5,5010.0)	0.005	0.926
PLT/10^9^·L^−1^	195.0(168.0,223.0)	101.0(62.5,158.0)	84.0(53.5,124.5)	<0.01	0.229
TBA/umol·L-1	22.0(7.0,37.0)	35.5(17.3,97.8)	47.0(16.0,108.3)	0.033	0.853
Cr/umol·L-1	59.0(51.0,70.0)	60.0(53.5,65.8)	66.0(60.5,74.3)	0.371	0.081
PT /s	11.2(10.6,11.7)	12.3(11.0,13.7)	12.8(11.2,14.7)	0.008	0.188
ANA	1:100(1:486,1:100)	1:320(1:1,000,1:100)	1:320(1:1000,1:100)	0.102	0.669
γ-globulin/%	24.0(18.0,28.8)	29.4(22.4,33.8)	32.0(25.6,35.0)	0.052	0.4
With ascites (%)	0(0.00)	0(0.00)	26(86.7)	–	–
With HE (%)	0(0.00)	0(0.00)	0(0.00)	–	–
APRI score	31.9(12.8,55.8)	69.9(35.8,185.4)	97.2(38.3,159.9)	0.004	0.697

*All clinical information in the table indicates data collected at the time closest to blood collection*.

*Median and quartile values are provided as Median (upper quartile, lower quartile), unless otherwise noted as n (%)*.

*P^a^ is the significant difference comparison between non-cirrhosis and compensated cirrhosis patients, P^b^ between compensated and decompensated cirrhosis patients*.

*ALT, alanine aminotransferase. AST, aspartate transaminase. ALP, alkaline phosphatase. GGT, γ-glutamyl transpeptidase. TBil, total bilirubin. DBil, direct bilirubin. IgA, immunoglobulin A. IgG, immunoglobulin G. IgM, immunoglobulin M. CHE, cholinesterase. INR, international normalized ratio. TC, total cholesterol. TG, triglyceride. ALB, albumin. WBC, white blood cell. PLT, platelet. TBA, total bile acid. Cr, creatinine. PT, prothrombin time. ANA, antinuclear. HE, hepatic encephalopathy. APRI, AST to PLT ratio index*.

### Patients With Cirrhosis Exhibited Different Anabolism and Metabolism Characteristics

Compared to non-cirrhotic AIH patients, those that developed into the cirrhosis stage (CC or DC) showed different characteristics, including sera enzymes, liver-synthesizing proteins, liver-metabolizing metabolites, and coagulation function (Table 1). When cirrhosis developed, significant decrease in albumin and white blood cell counts were observed. The coagulation function indices (prothrombin time and international normalized ratio) increased significantly, but were not significant between CC and DC groups. Bilirubin (TBiL and DBiL) increased significantly in the CC group compared to the NC group, indicating potential liver dysfunction of detoxication. Along with the evolving disease stages, cholinesterase decreased significantly. Together, these results indicated a gradual disturbance of liver functions such as anabolism and metabolism in the evolution of AIH.

### Liver Cirrhosis and Decompensation Stages Associated With Different Sera Metabolites

**Figure 1:**
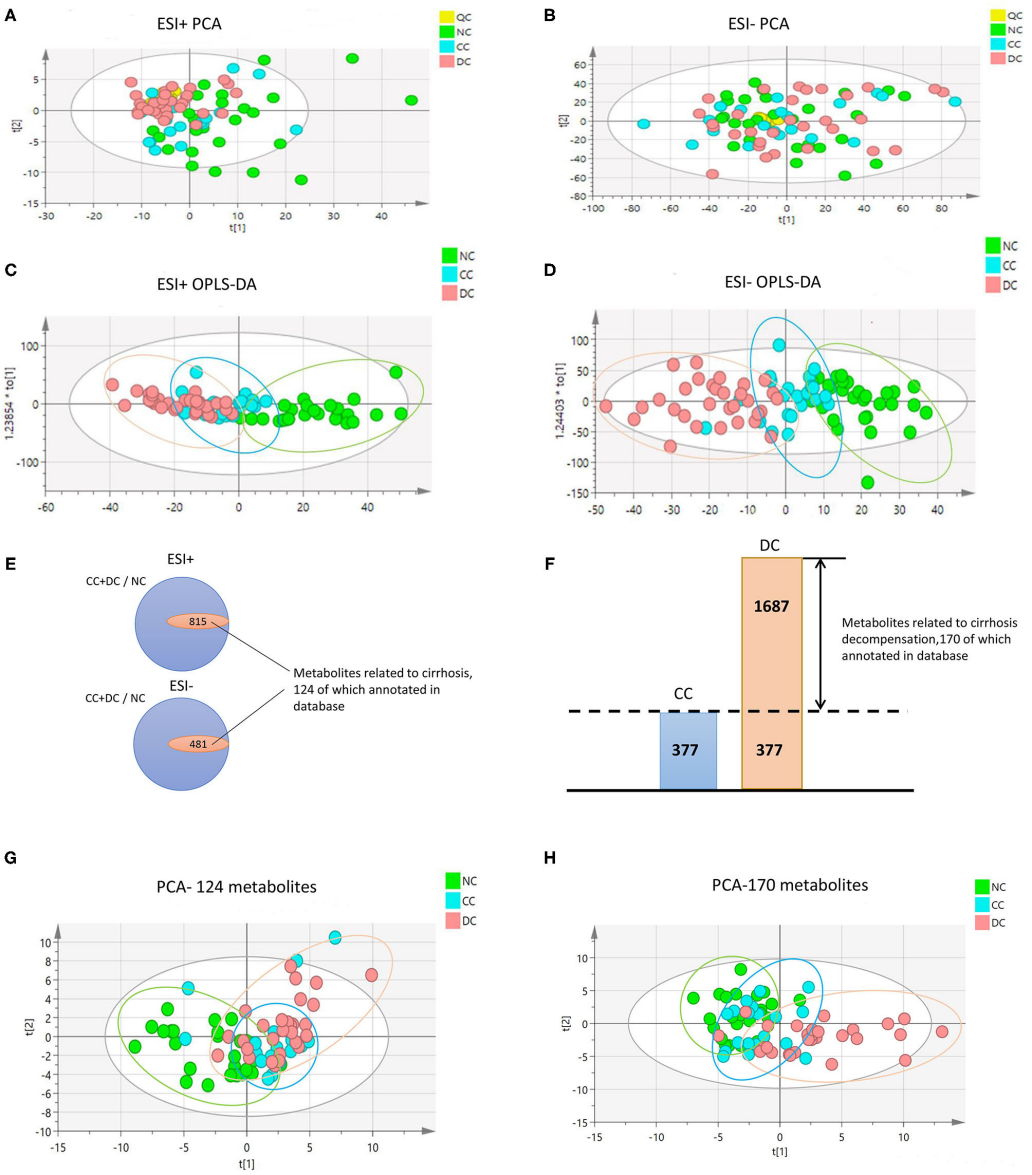
Metabolome profile for cirrhosis at different period. **(A–D)** are PCA and OPLS-DA with whole variables among NC, CC, and DC under positive and negative model. The great difference among NC, CC, and DC under OPLS-DA demonstrated the difference among groups. **(E,F)** Venn diagram. **(E)** The inner parts means the set of variables related to progression of cirrhosis, 124 of which annotated in database was metabolome feature for cirrhosis. **(F)** The part of DC was the set of variables related to decompensation, 170 of which annotated in database was metabolome feature for decompensation. **(G,H)** are PCA among NC, CC, and DC with metabolome feature for cirrhosis and decompensation. They showed that great discrimination of NC and DC respectively.

We observed significant alterations in the metabolomic profiles from either positive or negative modes of mass spectrometry, in different stages of AIH (Figures 1A–D). This indicates that the progression from NC to CC and DC was a major contributor for the alteration of the sera metabolome. To explore the underlying relationship between the evolving stages of AIH and the patterns of sera metabolite levels, we firstly obtained metabolic entities with significant difference (*P* < 0.05) between NC and CC, as well as CC vs. DC. The Venn diagram of these metabolic entities showed over 1,200 metabolites (including both positive and negative modes) that were associated with cirrhosis [(CC+DC) vs. NC]; while over 1,600 metabolites were solely associated with decompensation (DC vs. CC) (Figures 1E,F). We then identified 124 and 170 metabolites in these two sets of variables, respectively. Based on these metabolites, we found that the PCA plot of 124 cirrhosis-related metabolites showed an obvious separation of NC from either CC or DC, while CC and DC overlapped (Figure 1G). The PCA plot of the 170 decompensation-related metabolites revealed an evident separation of DC from either CC or NC, while CC and NC were located in the same zone (Figure 1H). These results indicate two distinct metabolic features associated with cirrhosis or decompensation stages. The fold changes of these annotated metabolites between the study groups are showed in Supplementary Figure 1.

### Cirrhosis-Associated Sera Metabolites Composed of a Fingerprint for AIH-Related Liver Cirrhosis

To reduce the dimension of the data set and find a unique metabolomic fingerprint of AIH-related liver cirrhosis, we computed the AUC and *P*-values of each of the 124 cirrhosis-related metabolites in differentiating cirrhosis (either CC or DC) from non-cirrhosis (NC). The resulting AUCs and associated *P*-values were used for a hierarchical cluster analysis and shown in a heat-map, indicating the highest relevant cluster (including 42 metabolites) with a highly significant association with cirrhosis [Supplementary Figure 2 (I)]. The AUC values in this cluster for cirrhosis ranged from 0.6786 to 0.7835 with significant *P*-values (Supplementary Tables 1, 3). Interestingly, the 42-metabolite cluster identified in Supplementary Figure 2 (I), the high AUC values observed in cirrhosis (either CC or DC) were maintained when patients were separated into CC or DC groups [Supplementary Figure 2(II,III)]. These results indicate a unique metabolome fingerprint (42 metabolites) among patients with cirrhosis.

**Figure 2:**
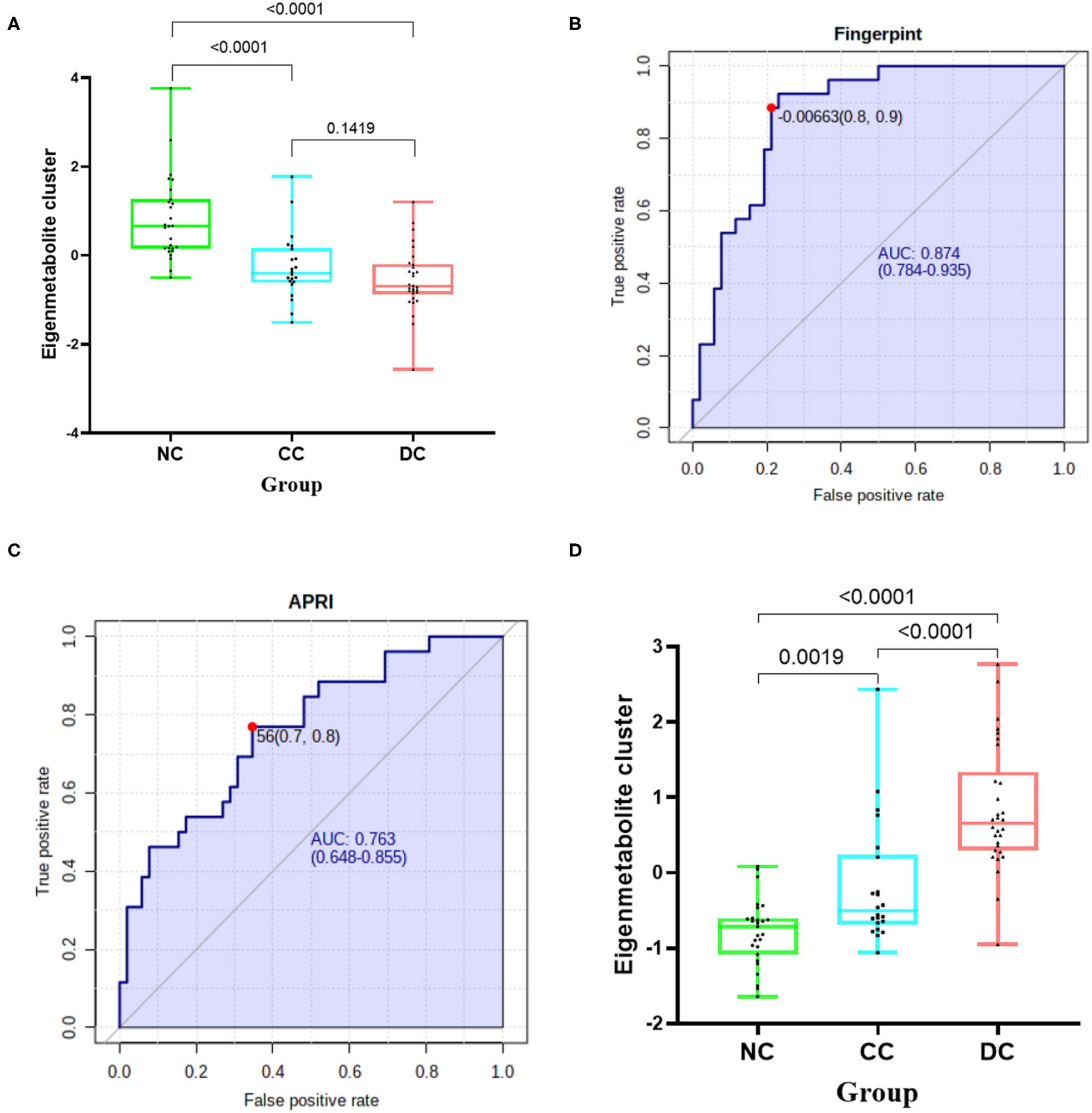
Identification of cirrhosis-related metabolome fingerprint. **(A)** Showed the decreasing trend of eigenmetabolite of metabolome fingerprint along with the progression of cirrhosis. **(B)** Demonstrated the cirrhosis-associated metabolic fingerprint (eigenmetabolite) revealed better capability to identify cirrhotic patients from non-cirrhotic ones(AUC:0.874). **(C)** The ROC curve of APRI in discriminating cirrhosis from non-cirrhotic AIH patients(AUC:0.763). **(D)** Showed that the increasing trend of eigenmetabolite of metabolome fingerprint from NC to CC and DC.

To further display the metabolome fingerprint trend in AIH development, we computed an eigenmetabolite (15) using PCA as a representative variable of the aforementioned 42-metabolite cluster. The eigenmetabolite showed progressively decreasing trend across the different stages of AIH and reached a minimum in DC (Figure 2A). Interestingly, there was a significant decrease of the eigenmetabolite in CC compared to NC, but no significance was observed between CC and DC. This trend supports the result that the 42 metabolites represented a metabolic fingerprint for AIH-related liver cirrhosis.

### Cirrhosis-Associated Metabolic Fingerprint Showed Better Diagnostic Performance in Discerning AIH Cirrhosis Than That of APRI

We found that the cirrhosis-associated metabolic fingerprint (eigenmetabolite) had better capability of identifying cirrhotic from non-cirrhotic patients than APRI (AUC values 0.874 vs. 0.763, Figures 2B,C). Collectively, these findings indicate that the 42-metabolite cluster can serve as a fingerprint of sera metabolites, which characteristically differentiated cirrhosis from non-cirrhosis.

### Decompensation-Associated Sera Metabolites Composed a Fingerprint for Differentiating Decompensation Stage

Similarly, for each of the 170 metabolites in decompensation-associated metabolome feature, we computed the AUC assessing the differentiation accuracy of each metabolite in decompensated cirrhosis from the compensation stage. The resulting AUCs and associated *P*-values were used for a hierarchical cluster analysis, which identified the highest relevant cluster including 47 metabolites with a highly significant association with cirrhosis (AUC for cirrhosis and significant *P* shown in [Supplementary Figure 3, Supplementary Table 2]). We then computed an eigenmetabolite by PCA, which is representative of the 47-metabolite cluster and correlated the eigenmetabolite with the progression of AIH, from NC to CC and DC (Figure 2D). The eigenmetabolite showed an increasing trend and significant differences (all *P* < 0.05) among NC, CC, and DC.

### Metabolic Fingerprints Indicating Loss of Liver Anabolic Functions

**Figure 3:**
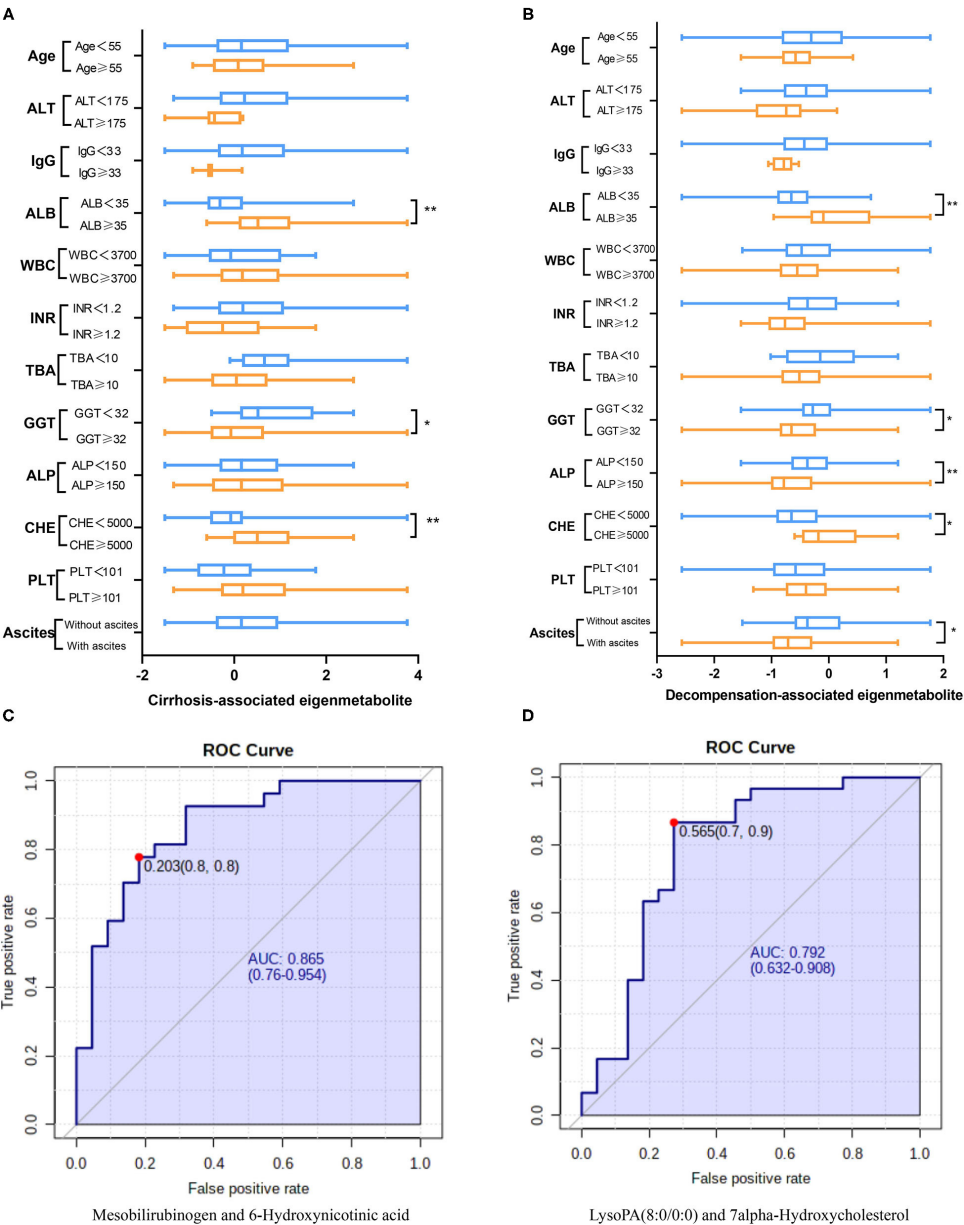
Clinical associations of the two metabolic fingerprints and the diagnostic ability of paired metabolites. **(A)** Comparison between clinical indices and the cirrhosis-associated fingerprint (eigenmetabolite) in NC and CC patients. **(B)** Comparison between clinical indices and the decompensation-associated fingerprint (eigenmetabolite) in CC and DC patients. **(C)** The diagnosis ability of intensity ratio between Mesobilirubinogen and 6-Hydroxynicotinic acid to differentiate NC and CC. **(D)** The diagnosis ability of intensity ratio between LysoPA(8:0/0:0) and 7alpha-Hydroxycholesterol to differentiate CC and DC(The units of each index are indicated in Table 1).

We further compared trends of the two metabolic fingerprints separately using clinically important indices (14, 17, 18). Cirrhosis-associated eigenmetabolite were significantly higher in NC and CC patients with high-albumin (Figure 3A), which indicates the loss of liver anabolic function in cirrhotic AIH patients. Albumin-associated scores may be useful to evaluate long-term prognosis in patients with autoimmune-related hepatic cirrhosis (19). Similarly, high level of eigenmetabolite was observed in patients with high cholinesterase. In some clinical indices, decompensation-associated metabolic fingerprint in CC and DC patients had significant alterations in their subgroups (Figure 3B). Notably, significant *P*-value also existed between patients with low-albumin and high-albumin indicating further loss of liver anabolic functions in decompensated cirrhotic patients. In addition, high level of alkaline phosphatase corelated with low level of decompensation-associated eigenmetabolite, indicating low anabolic liver function (20, 21). Besides, low levels of eigenmetabolite were observed in patients with high γ-glutamyl transpeptidase in cirrhosis and decompensated patients. Gamma-glutamyl transpeptidase and albumin were independent predictors of significant fibrosis (22, 23). In cirrhotic patients with ascites, low level of decompensation-associated eigenmetabolite was observed compared to non-ascites patients.

### Paired Metabolites Reveal Diagnostic and Prognostic Capability for AIH

From the cirrhosis-associated fingerprint, a metabolites pair—Mesobilirubinogen and 6-Hydroxynicotinic acid—was further screened out to construct a novel diagnostic parameter to identify AIH-related cirrhosis patients (Figure 3C). The diagnostic ability of such paired metabolites is based on the intensity ratio, which was 0.865 of AUC. The intensity ratio of paired metabolites can eliminate the differences between instruments and provide better methodology and good applicability for clinical use. Similarly, another metabolites pair—LysoPA(8:0/0:0) and 7alpha-Hydroxycholesterol—was screened out from the decompensation-associated fingerprint and achieved 0.792 of AUC in differentiating decompensated cirrhosis from non-decompensated ones (Figure 3D).

### Metabolic Fingerprints Have Potential Pathophysiological Significance in the Evolution of AIH Cirrhosis

**Figure 4:**
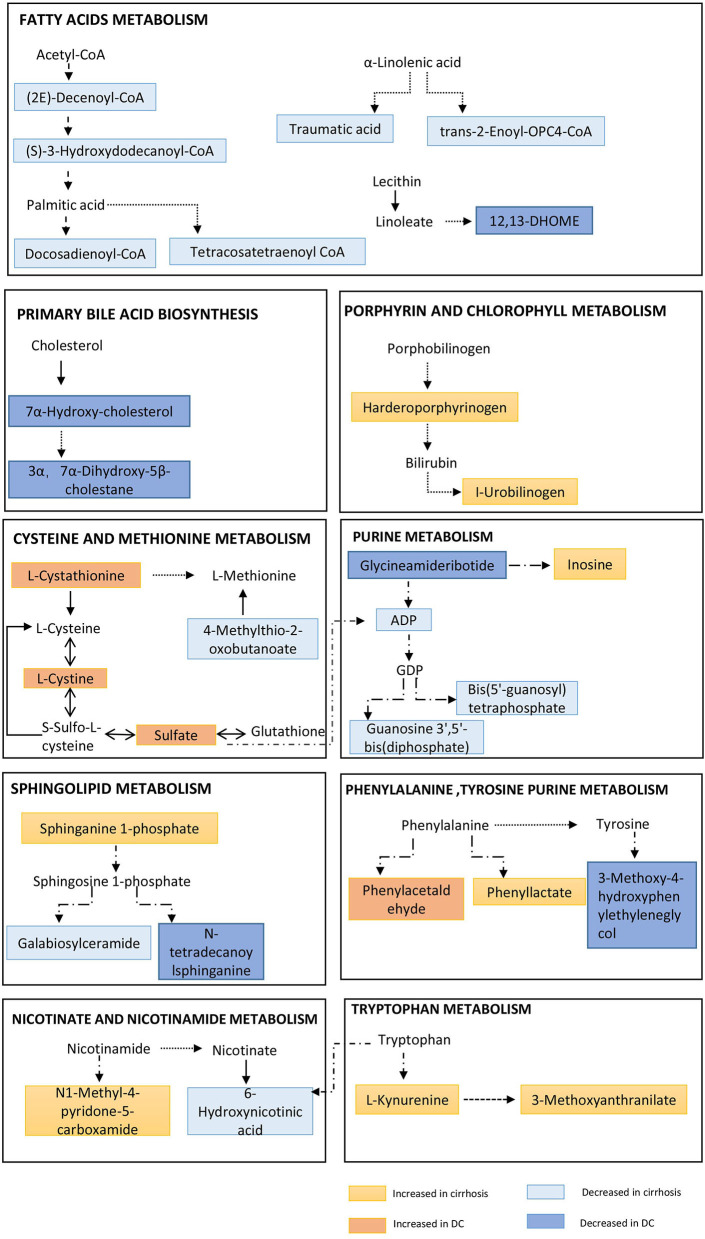
Pathway enrichment of metabolites in metabolome fingerprint. A typical feature is that a series of metabolites related to long-chain fatty acids (e.g., 12,13-DHOME, trans-2-Enoyl-OPC4-CoA and Docosadienoyl-CoA, etc.) are significantly reduced. And, a key feature of decompensated liver cirrhosis was observed to be used as energy supply by proteolysis (incomplete breakdown products of protein catabolism increased significantly in DC group).

Several energy sources and intermediary metabolites in the plasma of patients with liver cirrhosis changed significantly compared to non-cirrhosis patients, which indicates a particularly significant energy metabolism disorder. We found that the two metabolic fingerprints are obviously related to the energy supply related to the interference of metabolic pathways, especially increased lipolysis and proteolysis (Figure 4). These results indicate that abnormalities in metabolic fingerprints may be involved in the progression of the disease.

#### AIH Cirrhosis Associates With Increased Lipolysis

In the two metabolic fingerprints for different stages of AIH, a typical feature is that a series of metabolites related to long-chain fatty acids (e.g.,12,13-DHOME, trans-2-Enoyl-OPC4-CoA, Docosadienoyl-CoA, Tetrac-osatetraenoyl-CoA and (S)-3-Hydroxydodecanoyl-CoA, etc.) are significantly reduced (Supplementary Tables 1, 2). Increased lipolysis and suppressed lipogenesis strikingly promote the energy supplying flux by fatty acids.

#### AIH Cirrhosis Associates With Increased Proteolysis

Due to a large amount of energy expenditure, a key feature of decompensated liver cirrhosis was observed to be used as energy supply by proteolysis (Figure 4). Several proteolysis markers Glutaminylphenylalanine, Hydroxyprolyl-Tyrosine, and Glutaminyltryptophan, and incomplete breakdown products of protein catabolism, increased significantly in DC group compared to the NC or CC groups (Supplementary Table 3).

## Discussion

This paper is the first study utilizing untargeted and whole-spectrum metabolomics approach in sera samples of AIH patients without cirrhosis or with cirrhosis at either compensation or decompensation stages. We identified two metabolic fingerprints for different stages of AIH. The fingerprint of metabolites related to liver cirrhosis includes 42 metabolites, reflecting the characteristic metabolic changes in the process of AIH progression to liver cirrhosis, without distinguishing between compensatory and decompensated states. Compared to non-invasive liver cirrhosis evaluation index APRI, liver cirrhosis-related metabolite fingerprints have a better diagnostic ability to distinguish between non-cirrhosis and liver cirrhosis. The metabolic fingerprint related to decompensation includes 47 metabolites, reflecting the characteristic metabolic changes from cirrhosis to the decompensated stage. These metabolic characteristics provide a new perspective to understand the underlying mechanism of AIH disease progression. Specifically, the analysis of pathway enrichment by combining two metabolic fingerprints indicates that AIH progresses to liver cirrhosis and decompensated stage, which is manifested by obvious disturbances in metabolic processes related to energy supply, and nutrient metabolism is the basic characteristic of decomposition and consumption. These metabolic characteristics are further strengthened from compensated cirrhosis to decompensated cirrhosis.

AIH-related immune response may be caused by the autoantigens of initial CD4+ T cells (24), which further forms continuous immune response and inflammatory reaction. There are many studies on the relationship between immunology and metabolism (25–27), but little is known about the metabolic adaptation and characteristics of AIH disease progression. AIH is mainly characterized by liver interface inflammation. As the disease progresses, it produces energy dependence, leading to energy competition between the immune system and other programs in the body. At the same time, the energy supply in the body is obviously disordered, and nutrients (such as lipids and proteins) are consumed in large quantities (7),which manifests as increased lipolysis and proteolysis. In this study, we found that a group of pathways involved in energy metabolism constituting the metabonomic characteristics of AIH. L-kynurenine and its further breakdown products carry out diverse biological functions, including dilating blood vessels during inflammation and regulating the immune response (28, 29). The elevation of L-kynurenine is associated with the presence of inflammation and energy expenditure (7).

We also observed significant decrease of 7alpha-hydroxycholesterol (the catalyzing product of Cyp7a1) in decompensated autoimmune hepatic cirrhosis (30). Previous studies have shown that Cyp7a1 is positively associated with liver inflammation (31, 32). Cyp7a1 is the rate-limiting enzyme in the “classic pathway” of bile acids synthesis. The reduction of bile acid synthesis will inevitably cause the dynamic balance of enterohepatic circulation, which will lead to persistent inflammation in the liver (33). In addition, bile acids can help emulsify fat, enhance lipolysis, and improve the solubility of lipids by forming mixed micelles, promote the absorption of lipids in the intestine, and improve lipolysis. And, the interaction between bile acids and intestinal microbiota is not unidirectional. It is necessary to study the intestinal bacteria of AIH patients to elucidate the potential impact of gut microbial on bile acids metabolism and its further effects on liver immune and metabolic microenvironments. Interestingly, the metabolites related to long-chain fatty acids in the metabolic fingerprint of cirrhosis are all reduced, which indicate that β-oxidation of fatty acids is enhanced, and the body needs to consume a lot of energy for immune response and immune cell activation and proliferation (34). There are many proteins with incomplete catabolism in the fingerprint of decompensated metabolism. These findings imply that proteolysis may increase due to energy requirements. In previous reports, the skeletal muscles of rats have a significant accumulation of sphinganine and sphingosine after exercise, which may be related to the increase in energy requirements. Furthermore, ceramide promotes apoptosis whereas sphingosine-1-phosphate can inhibit apoptosis and induce cell growth. So, the reaction will tend to transform to sphingosine. Therefore, Galabiosyl ceramide and N-tetradecanoyl sphinganine will decrease (35).

Based on the favorable aspects of metabolic reprogramming mechanisms of AIH, we can explore the utility of characteristic metabolites to facilitate diagnosis or monitoring of disease progression. We therefore found that the intensity ratio of a pair of metabolites [Mesobilirubinogen and 6-Hydroxynicotinic acid] can be applied to recognizing cirrhosis in AIH patients; furthermore, the paired metabolites [LysoPA(8:0/0:0) and 7alpha-Hydroxycholesterol] can predict the development of decompensated cirrhosis for AIH. Since the intensity ratio of paired metabolites can eliminate the system bias of instruments in different laboratories, these metabolite pairs can be much easier to be applied in clinical settings. Compared with targeted quantitation of metabolites by mass spectrometry, the intensity ratio of paired metabolites is much cheaper and simpler (36).

In summary, liver metabolic dysfunction featuring enhanced lipolysis and increased proteolysis, may play an important role in the pathogenesis and progression of liver cirrhosis in AIH patients. The established metabolic fingerprint profile related to liver cirrhosis and decompensation can be used as a resource for metabolic adaptation and metabolic reprogramming of AIH, and provide guidance for future clinical prognosis, mechanisms, and new treatment research. Furthermore, this finding suggests that metabolic reprogramming may be a new direction in treating AIH.

## Data Availability Statement

The original contributions presented in the study are included in the article/Supplementary Material, further inquiries can be directed to the corresponding authors.

## Ethics Statement

The studies involving human participants were reviewed and approved by Medical Ethics Committee of Fifth Medical Center of Chinese PLA General Hospital. The patients/participants provided their written informed consent to participate in this study.

## Author Contributions

J-bW, Y-cY, and Z-sZ were responsible for the study concept and design. S-sL, JJ, S-sC, YH, ZS, G-zS, and XH performed sample collection, conducted LC-MS data analysis, and manuscript preparation. S-sL and J-bW drafted the manuscript. Z-tZ, H-zZ, Z-sZ, and Y-cY reviewed and modified the manuscript. MN helped with data analysis. All authors contributed to the article and approved the submitted version.

## Conflict of Interest

The authors declare that the research was conducted in the absence of any commercial or financial relationships that could be construed as a potential conflict of interest.
