# Supplementary material for: Metabolomic Signatures of Autoimmune Hepatitis in the Development of Cirrhosis
Source: Front Med (Lausanne). 2021 Mar 12;8:644376. doi: 10.3389/fmed.2021.644376 (PMC7994277; doi:10.3389/fmed.2021.644376)
Supplement: Supplementary file 1 [file Data_Sheet_1.ZIP › Supplementary information.docx]

**Supplementary information**

**Fig. S1 Metabolic feature for cirrhosis and decompensation.**

A, Cleveland plots of 124 metabolites-metabolome feature for cirrhosis. B, Cleveland plots of 170 metabolites-metabolome feature for decompensation. The whole set of metabolites was ranked by FC in CC/NC and DC/NC. The bottom one of both A and B, zooming on top fifty metabolites in two comparisons.

**Fig. S2 Hierarchical cluster analysis of cirrhosis-related metabolome fingerprint.**

(Ⅰ), hierarchical cluster analysis of AUC and P differentiating cirrhosis to non-cirrhosis. (Ⅱ), corresponding metabolites in accessing CC relative to NC. (Ⅲ), corresponding metabolites in accessing DC relative to NC.

**Fig. S3 Hierarchical cluster analysis of decompensation-related metabolome fingerprint.**

hierarchical cluster analysis of AUC and P differentiating DC to CC.

**Table S1 The detailed information about 42 metabolites in metabolome fingerprint for cirrhosis.**

| **No.** | **Mode** | **MZ** | **RT** | **Name** | **HMDB** | **Formula** | **Cirrhosis vs Non-cirrhosis** | | | |  | | **Compensation cirrhosis vs Non-cirrhosis** | | | | | | |  | | | **Decompensation cirrhosis vs Non-cirrhosis** | | | | | | |  |  |
| --- | --- | --- | --- | --- | --- | --- | --- | --- | --- | --- | --- | --- | --- | --- | --- | --- | --- | --- | --- | --- | --- | --- | --- | --- | --- | --- | --- | --- | --- | --- | --- |
|  |  |  |  |  |  |  | **AUC** | **FC** | **log2(FC)** | **P** | |  | | **AUC** | **FC** | | **log2(FC)** | | **P** | |  | | | **AUC** | **FC** | | | **log2(FC)** | | **P** |  |
| 1 | ESI+ | 986.2754 | 11.48 | trans-2-Enoyl-  OPC4-CoA | HMDB0011120 | C35H54N7O18P3S | 0.774 | 0.361 | -1.471 | 0.000 | | 0.737 | | | 0.441 | -1.180 | | 0.015 | | | | 0.801 | | | | 0.302 | -1.729 | | 0.000 | | |
| 2 | ESI+ | 1110.4253 | 11.55 | Tetracosatetraenoyl  CoA | HMDB0006516 | C45H74N7O17P3S | 0.776 | 0.415 | -1.267 | 0.000 | | 0.758 | | | 0.476 | -1.071 | | 0.023 | | | | 0.789 | | | | 0.371 | -1.431 | | 0.001 | | |
| 3 | ESI+ | 161.0765 | 1.71 | Pimelic acid | HMDB0000857 | C7H12O4 | 0.783 | 0.368 | -1.441 | 0.002 | | 0.788 | | | 0.280 | -1.838 | | 0.002 | | | | 0.780 | | | | 0.433 | -1.207 | | 0.027 | | |
| 4 | ESI+ | 966.2686 | 11.58 | (S)-3-Hydroxydo  decanoyl-CoA | HMDB0003936 | C33H58N7O18P3S | 0.758 | 0.375 | -1.414 | 0.003 | | 0.768 | | | 0.378 | -1.405 | | 0.041 | | | | 0.751 | | | | 0.373 | -1.422 | | 0.015 | | |
| 5 | ESI- | 415.2058 | 4.72 | Cyclic Phosphatidic  acid(18:2) | HMDB0007007 | C21H37O6P | 0.755 | 3.375 | 1.755 | 0.005 | | 0.688 | | | 2.668 | 1.416 | | 0.052 | | | | 0.803 | | | | 3.876 | 1.955 | | 0.001 | | |
| 6 | ESI+ | 881.748 | 11.53 | TG(16:1(9Z)/18:0/20:4  (5Z,8Z,11Z,14Z)) | HMDB0005426 | C57H100O6 | 0.745 | 0.439 | -1.186 | 0.000 | | 0.692 | | | 0.555 | -0.848 | | 0.027 | | | | 0.784 | | | | 0.354 | -1.497 | | 0.000 | | |
| 7 | ESI+ | 154.0486 | 1.39 | 2-(hydroxyamino)  benzoic acid | HMDB0164041 | C7H7NO3 | 0.743 | 2.778 | 1.474 | 0.003 | | 0.657 | | | 1.747 | 0.805 | | 0.047 | | | | 0.806 | | | | 3.533 | 1.821 | | 0.000 | | |
| 8 | ESI- | 181.9656 | 1.06 | Se-Methylselenocysteine | HMDB0004113 | C4H9NO2Se | 0.750 | 0.601 | -0.734 | 0.001 | | 0.691 | | | 0.712 | -0.491 | | 0.077 | | | | 0.791 | | | | 0.523 | -0.935 | | 0.000 | | |
| 9 | ESI+ | 920.2093 | 9.97 | (2E)-Decenoyl-  CoA | HMDB0003948 | C31H52N7O17P3S | 0.738 | 0.419 | -1.255 | 0.000 | | 0.732 | | | 0.430 | -1.219 | | 0.009 | | | | 0.742 | | | | 0.412 | -1.281 | | 0.002 | | |
| 10 | ESI- | 426.0212 | 1.18 | ADP | HMDB0001341 | C10H15N5O10P2 | 0.735 | 0.610 | -0.712 | 0.032 | | 0.672 | | | 0.632 | -0.662 | | 0.041 | | | | 0.779 | | | | 0.595 | -0.749 | | 0.077 | | |
| 11 | ESI- | 117.0319 | 1.26 | xi-2,3-Octadiene-  5,7-diyn-1-ol | HMDB0040549 | C8H6O | 0.725 | 2.219 | 1.150 | 0.035 | | 0.669 | | | 1.580 | 0.660 | | 0.051 | | | | 0.765 | | | | 2.673 | 1.418 | | 0.022 | | |
| 12 | ESI- | 389.0879 | 3.62 | Dopaxanthin | HMDB0012221 | C18H18N2O8 | 0.720 | 4.648 | 2.217 | 0.045 | | 0.646 | | | 5.867 | 2.553 | | 0.044 | | | | 0.773 | | | | 3.783 | 1.920 | | 0.037 | | |
| 13 | ESI+ | 1086.4249 | 11.59 | Docosadienoyl-CoA | HMDB0062220 | C43H74N7O17P3S | 0.706 | 0.482 | -1.051 | 0.001 | | 0.721 | | | 0.470 | -1.091 | | 0.012 | | | | 0.695 | | | | 0.492 | -1.023 | | 0.007 | | |
| 14 | ESI- | 138.0197 | 5 | 6-Hydroxynicotinic acid | HMDB0002658 | C6H5NO3 | 0.705 | 0.517 | -0.952 | 0.001 | | 0.702 | | | 0.527 | -0.925 | | 0.027 | | | | 0.707 | | | | 0.510 | -0.972 | | 0.006 | | |
| 15 | ESI+ | 229.141 | 9.04 | Traumatic acid | HMDB0000933 | C12H20O4 | 0.709 | 0.506 | -0.983 | 0.002 | | 0.714 | | | 0.523 | -0.936 | | 0.032 | | | | 0.705 | | | | 0.494 | -1.018 | | 0.005 | | |
| 16 | ESI+ | 353.0329 | 1.05 | [4-(3,7-dihydroxy-4-oxo-3,4-  dihydro-2H-1-  benzopyran-2-yl)phenyl]  oxidanesulfonic acid | HMDB0151702 | C15H12O8S | 0.714 | 0.649 | -0.623 | 0.001 | | 0.709 | | | 0.671 | -0.575 | | 0.023 | | | | 0.717 | | | | 0.633 | -0.660 | | 0.002 | | |
| 17 | ESI+ | 601.4737 | 21.58 | DG(15:0/20:5  (5Z,8Z,11Z,14Z,17Z)/0:0) | HMDB0007085 | C38H64O5 | 0.716 | 0.621 | -0.687 | 0.006 | | 0.684 | | | 0.628 | -0.671 | | 0.017 | | | | 0.740 | | | | 0.616 | -0.699 | | 0.028 | | |
| 18 | ESI+ | 257.1172 | 11.62 | 1-(beta-D-Ribofuranosyl)-  1,4-dihydronico  tinamide | HMDB0011648 | C11H16N2O5 | 0.721 | 0.476 | -1.072 | 0.003 | | 0.678 | | | 0.556 | -0.847 | | 0.086 | | | | 0.752 | | | | 0.417 | -1.262 | | 0.007 | | |
| 19 | ESI+ | 153.065 | 1.48 | N1-Methyl-4-  pyridone-3-  carboxamide | HMDB0004194 | C7H8N2O2 | 0.739 | 2.340 | 1.227 | 0.017 | | 0.709 | | | 1.701 | 0.766 | | 0.066 | | | | 0.762 | | | | 2.810 | 1.490 | | 0.007 | | |
| 20 | ESI- | 124.9925 | 1.21 | 2-Hydroxyethanesulfonate | HMDB0003903 | C2H6O4S | 0.733 | 2.595 | 1.376 | 0.014 | | 0.727 | | | 1.687 | 0.754 | | 0.008 | | | | 0.736 | | | | 3.240 | 1.696 | | 0.006 | | |
| 21 | ESI+ | 1105.5616 | 11.53 | 3,4,5-trihydroxy-6-{[(4Z,10Z,12E,14E,19Z)-7,16,17-trihydroxydocosa-  4,8,10,12,14,19-hexaenoyl]oxy}oxane-2-carboxylic acid | HMDB0183070 | C28H40O11 | 0.726 | 0.447 | -1.161 | 0.000 | | 0.709 | | | 0.514 | -0.960 | | 0.026 | | | | 0.740 | | | | 0.398 | -1.329 | | 0.001 | | |
| 22 | ESI+ | 1097.6746 | 11.82 | Ganglioside GM3  (d18:1/12:0) | HMDB0004842 | C53H96N2O21 | 0.729 | 0.470 | -1.090 | 0.001 | | 0.699 | | | 0.506 | -0.984 | | 0.019 | | | | 0.752 | | | | 0.444 | -1.172 | | 0.003 | | |
| 23 | ESI+ | 593.3291 | 8.14 | Mesobilirubinogen | HMDB0001898 | C33H44N4O6 | 0.722 | 4.792 | 2.261 | 0.012 | | 0.790 | | | 3.512 | 1.812 | | 0.001 | | | | 0.673 | | | | 5.731 | 2.519 | | 0.013 | | |
| 24 | ESI+ | 382.2585 | 10.53 | Sphinganine 1-  phosphate | HMDB0001383 | C18H40NO5P | 0.725 | 2.439 | 1.286 | 0.013 | | 0.694 | | | 2.023 | 1.016 | | 0.010 | | | | 0.748 | | | | 2.744 | 1.456 | | 0.013 | | |
| 25 | ESI+ | 615.307 | 11.06 | Harderopor  phyrinogen | HMDB0002160 | C35H42N4O6 | 0.697 | 2.720 | 1.444 | 0.007 | | 0.687 | | | 2.765 | 1.467 | | 0.018 | | | | 0.705 | | | | 2.687 | 1.426 | | 0.007 | | |
| 26 | ESI- | 498.2878 | 6.97 | Taurodeoxycholic acid | HMDB0000896 | C26H45NO6S | 0.701 | 3.451 | 1.787 | 0.011 | | 0.699 | | | 2.589 | 1.372 | | 0.076 | | | | 0.703 | | | | 4.063 | 2.023 | | 0.004 | | |
| 27 | ESI+ | 603.9626 | 9.96 | Guanosine 3',5'-bis  (diphosphate) | HMDB0059638 | C10H17N5O17P4 | 0.696 | 0.410 | -1.286 | 0.001 | | 0.704 | | | 0.396 | -1.337 | | 0.016 | | | | 0.690 | | | | 0.421 | -1.249 | | 0.008 | | |
| 28 | ESI+ | 869.0432 | 11.59 | Bis(5'-guanosyl)  tetraphosphate | HMDB0001340 | C20H28N10O21P4 | 0.697 | 0.422 | -1.243 | 0.001 | | 0.668 | | | 0.455 | -1.137 | | 0.032 | | | | 0.717 | | | | 0.399 | -1.326 | | 0.005 | | |
| 29 | ESI+ | 209.0923 | 1.86 | L-Kynurenine | HMDB0000684 | C10H12N2O3 | 0.698 | 1.515 | 0.600 | 0.003 | | 0.751 | | | 1.589 | 0.668 | | 0.002 | | | | 0.659 | | | | 1.462 | 0.547 | | 0.016 | | |
| 30 | ESI+ | 277.0228 | 1.23 | 6-Phosphogluconic  acid | HMDB0001316 | C6H13O10P | 0.692 | 0.639 | -0.647 | 0.004 | | 0.618 | | | 0.737 | -0.441 | | 0.105 | | | | 0.746 | | | | 0.566 | -0.820 | | 0.003 | | |
| 31 | ESI- | 165.0556 | 1.29 | D-Phenyllactic acid | HMDB0000563 | C9H10O3 | 0.691 | 1.893 | 0.921 | 0.008 | | 0.580 | | | 1.303 | 0.382 | | 0.182 | | | | 0.770 | | | | 2.312 | 1.209 | | 0.001 | | |
| 32 | ESI+ | 1164.8136 | 12.53 | Trihexosylceramide  (d18:1/26:1(17Z)) | HMDB0004884 | C62H117NO18 | 0.685 | 0.558 | -0.841 | 0.011 | | 0.652 | | | 0.673 | -0.571 | | 0.209 | | | | 0.710 | | | | 0.474 | -1.076 | | 0.013 | | |
| 33 | ESI- | 267.0735 | 1.24 | Inosine | HMDB0000195 | C10H12N4O5 | 0.684 | 2.235 | 1.161 | 0.010 | | 0.614 | | | 1.818 | 0.863 | | 0.031 | | | | 0.733 | | | | 2.531 | 1.340 | | 0.007 | | |
| 34 | ESI+ | 616.3157 | 11.03 | Paromomycin | HMDB0015490 | C23H45N5O14 | 0.687 | 3.668 | 1.875 | 0.012 | | 0.650 | | | 3.692 | 1.884 | | 0.047 | | | | 0.714 | | | | 3.650 | 1.868 | | 0.002 | | |
| 35 | ESI+ | 149.0235 | 6.79 | 2-Oxo-4-methyl  thiobutanoic acid | HMDB0001553 | C5H8O3S | 0.680 | 0.488 | -1.035 | 0.004 | | 0.689 | | | 0.475 | -1.075 | | 0.036 | | | | 0.674 | | | | 0.498 | -1.006 | | 0.022 | | |
| 36 | ESI- | 1000.7324 | 22.08 | Galabiosylceramide  (d18:1/26:0) | HMDB0004839 | C56H107NO13 | 0.679 | 0.403 | -1.313 | 0.001 | | 0.669 | | | 0.390 | -1.358 | | 0.022 | | | | 0.685 | | | | 0.411 | -1.282 | | 0.012 | | |
| 37 | ESI- | 545.041 | 1.23 | dTDP-4-dehydro-6-  deoxy-alpha-D-glucose | HMDB0001399 | C16H24N2O15P2 | 0.716 | 0.491 | -1.026 | 0.018 | | 0.741 | | | 0.379 | -1.401 | | 0.013 | | | | 0.699 | | | | 0.571 | -0.809 | | 0.112 | | |
| 38 | ESI- | 499.2926 | 7.06 | Threonyl-prolyl-  arginyl-lysine | HMDB0005776 | C21H40N8O6 | 0.715 | 5.757 | 2.525 | 0.025 | | 0.727 | | | 5.178 | 2.373 | | 0.049 | | | | 0.706 | | | | 6.168 | 2.625 | | 0.019 | | |
| 39 | ESI+ | 60.0805 | 1.12 | Trimethylamine | HMDB0000906 | C3H9N | 0.701 | 1.704 | 0.769 | 0.019 | | 0.662 | | | 1.370 | 0.454 | | 0.046 | | | | 0.730 | | | | 1.949 | 0.963 | | 0.012 | | |
| 40 | ESI+ | 168.0631 | 1.42 | 3-Methoxy  anthranilate | HMDB0060374 | C8H9NO3 | 0.702 | 1.969 | 0.978 | 0.020 | | 0.652 | | | 1.483 | 0.569 | | 0.031 | | | | 0.738 | | | | 2.325 | 1.218 | | 0.011 | | |
| 41 | ESI- | 99.0453 | 1.14 | 2-methylbut-3-enoic acid | HMDB0130837 | C5H8O2 | 0.696 | 1.935 | 0.953 | 0.021 | | 0.657 | | | 2.215 | 1.147 | | 0.033 | | | | 0.724 | | | | 1.737 | 0.797 | | 0.003 | | |
| 42 | ESI- | 87.0454 | 1.2 | Butyric acid | HMDB0000039 | C4H8O2 | 0.705 | 2.507 | 1.326 | 0.031 | | 0.652 | | | 1.593 | 0.672 | | 0.056 | | | | 0.743 | | | | 3.156 | 1.658 | | 0.014 | | |

**Table S2** **The detailed information about 47 metabolites in metabolome fingerprint for decompensation.**

| **No.** | **Mode** | **MZ** | **RT** | **Name** | **HMDB** | **Formula** | **Compensation cirrhosis vs Non-cirrhosis** | | | |  | **Decompensation cirrhosis vs Non-cirrhosis** | | | |
| --- | --- | --- | --- | --- | --- | --- | --- | --- | --- | --- | --- | --- | --- | --- | --- |
|  |  |  |  |  |  |  | **AUC** | **FC** | **log2(FC)** | **P** |  | **AUC** | **FC** | **log2(FC)** | **P** |
| 1 | ESI- | 299.1027 | 4.2 | L-Cystathionine | HMDB0033142 | C17H16O5 | 0.588 | 3.300 | 1.722 | 0.185 |  | 0.729 | 2.858 | 1.515 | 0.043 |
| 2 | ESI- | 243.0774 | 1.45 | Biotin | HMDB0000030 | C10H16N2O3S | 0.552 | 1.283 | 0.360 | 0.432 |  | 0.725 | 7.039 | 2.815 | 0.048 |
| 3 | ESI- | 119.0488 | 1.23 | Phenylacetaldehyde | HMDB0006236 | C8H8O | 0.619 | 1.412 | 0.497 | 0.265 |  | 0.751 | 1.985 | 0.989 | 0.040 |
| 4 | ESI+ | 130.9651 | 8.47 | 2,2-dichloroethane-  1,1-diol | HMDB0062193 | C2H4Cl2O2 | 0.581 | 0.919 | -0.122 | 0.763 |  | 0.753 | 0.512 | -0.966 | 0.016 |
| 5 | ESI+ | 405.3709 | 20.82 | 3a,7a-Dihydroxy-5b-cholestane | HMDB0006893 | C27H48O2 | 0.582 | 1.106 | 0.145 | 0.828 |  | 0.754 | 0.389 | -1.364 | 0.025 |
| 6 | ESI- | 117.0319 | 1.26 | xi-2,3-Octadiene-5,7-  diyn-1-ol | HMDB0040549 | C8H6O | 0.669 | 1.580 | 0.660 | 0.051 |  | 0.765 | 2.673 | 1.418 | 0.022 |
| 7 | ESI+ | 295.1287 | 3.99 | Hydroxyprolyl-Tyrosine | HMDB0028875 | C14H18N2O5 | 0.588 | 1.239 | 0.309 | 0.148 |  | 0.747 | 1.448 | 0.534 | 0.001 |
| 8 | ESI+ | 277.0228 | 1.23 | 6-Phosphogluconic acid | HMDB0001316 | C6H13O10P | 0.618 | 0.737 | -0.441 | 0.105 |  | 0.746 | 0.566 | -0.820 | 0.003 |
| 9 | ESI+ | 514.2905 | 12.63 | Sulfolithocholylglycine | HMDB0002639 | C26H43NO7S | 0.567 | 0.924 | -0.114 | 0.680 |  | 0.742 | 0.555 | -0.849 | 0.000 |
| 10 | ESI+ | 153.065 | 1.48 | N1-Methyl-4-pyridone-3-carboxamide | HMDB0004194 | C7H8N2O2 | 0.709 | 1.701 | 0.766 | 0.066 |  | 0.762 | 2.810 | 1.490 | 0.007 |
| 11 | ESI- | 812.6602 | 1.04 | PE(24:1(15Z)/P-18:0) | HMDB0009775 | C47H92NO7P | 0.553 | 0.856 | -0.225 | 0.274 |  | 0.758 | 0.658 | -0.604 | 0.004 |
| 12 | ESI+ | 112.0856 | 13.02 | Histamine | HMDB0000870 | C5H9N3 | 0.650 | 0.917 | -0.124 | 0.768 |  | 0.754 | 0.486 | -1.041 | 0.000 |
| 13 | ESI+ | 257.1172 | 11.62 | 1-(beta-D-Ribofuranosyl)-  1,4-dihydronicotinamide | HMDB0011648 | C11H16N2O5 | 0.678 | 0.556 | -0.847 | 0.086 |  | 0.752 | 0.417 | -1.262 | 0.007 |
| 14 | ESI+ | 1164.8136 | 12.53 | Trihexosylceramide  (d18:1/26:1(17Z)) | HMDB0004884 | C62H117NO18 | 0.652 | 0.673 | -0.571 | 0.209 |  | 0.710 | 0.474 | -1.076 | 0.013 |
| 15 | ESI- | 127.0754 | 1.24 | 3-Hydroxy-3-methy  lcyclohexan-1-one | HMDB0165095 | C7H12O2 | 0.566 | 1.277 | 0.352 | 0.320 |  | 0.710 | 2.439 | 1.286 | 0.013 |
| 16 | ESI+ | 100.0757 | 1.89 | (2R)-2-Hydroxy-  2-methylbutanenitrile | HMDB0011749 | C5H9NO | 0.648 | 2.008 | 1.006 | 0.062 |  | 0.709 | 2.509 | 1.327 | 0.024 |
| 17 | ESI- | 559.3362 | 18.08 | (3,4-dihydroxyphenyl)methyl  7-methyloctanoate | HMDB0179992 | C16H24O4 | 0.613 | 0.672 | -0.574 | 0.198 |  | 0.710 | 0.499 | -1.003 | 0.023 |
| 18 | ESI+ | 185.0775 | 14.81 | 3-Methoxy-4-hydroxypheny  lethyleneglycol | HMDB0001490 | C9H12O4 | 0.658 | 0.718 | -0.478 | 0.070 |  | 0.714 | 0.649 | -0.623 | 0.020 |
| 19 | ESI+ | 459.2485 | 15.03 | N-tetradecanoylsphinganine | HMDB0002504 | C23H38O7S | 0.549 | 0.935 | -0.097 | 0.449 |  | 0.720 | 0.830 | -0.270 | 0.025 |
| 20 | ESI+ | 189.0865 | 1.26 | N-Acetylglutamine | HMDB0006029 | C7H12N2O4 | 0.525 | 0.878 | -0.188 | 0.425 |  | 0.720 | 0.608 | -0.717 | 0.008 |
| 21 | ESI- | 350.8962 | 1.11 | {[(8E)-10,15-dioxoheptadeca-  8,16-dien-11,13-diyn-7-yl]oxy}  sulfonic acid | HMDB0165270 | C17H20O6S | 0.545 | 1.073 | 0.102 | 0.730 |  | 0.720 | 1.706 | 0.770 | 0.008 |
| 22 | ESI- | 599.4635 | 21.33 | DG(15:0/20:5  (5Z,8Z,11Z,14Z,17Z)/0:0) | HMDB0007085 | C38H64O5 | 0.671 | 0.534 | -0.905 | 0.089 |  | 0.721 | 0.410 | -1.286 | 0.013 |
| 23 | ESI- | 153.0203 | 1.26 | 2,4,6-trihydroxy  benzaldehyde | HMDB0125090 | C7H6O4 | 0.549 | 1.595 | 0.674 | 0.215 |  | 0.720 | 2.712 | 1.439 | 0.013 |
| 24 | ESI- | 243.0618 | 1.2 | Pseudouridine | HMDB0000767 | C9H12N2O6 | 0.585 | 1.137 | 0.185 | 0.320 |  | 0.724 | 1.394 | 0.479 | 0.011 |
| 25 | ESI+ | 337.0613 | 1.37 | 3-hydroxy-4-methoxy-5-(3,4,5-  trihydroxybenzoyloxy)  benzoic acid | HMDB0128333 | C15H12O9 | 0.586 | 0.890 | -0.169 | 0.288 |  | 0.717 | 0.772 | -0.373 | 0.003 |
| 26 | ESI- | 360.1275 | 1.17 | Glutaminylphenylalanine | HMDB0028804 | C14H19N3O4 | 0.545 | 1.080 | 0.110 | 0.607 |  | 0.716 | 1.494 | 0.580 | 0.003 |
| 27 | ESI+ | 147.0436 | 1.32 | Coumarin | HMDB0135619 | C7H8O2 | 0.662 | 1.208 | 0.272 | 0.053 |  | 0.719 | 1.338 | 0.420 | 0.002 |
| 28 | ESI+ | 287.0556 | 0.98 | Glycineamideribotide | HMDB0002022 | C7H15N2O8P | 0.636 | 0.815 | -0.295 | 0.084 |  | 0.717 | 0.692 | -0.531 | 0.001 |
| 29 | ESI+ | 316.0829 | 1.21 | 2-({hydroxy[2-(2-hydroxybenzoyloxy)phenyl]  methylidene}amino)acetic acid | HMDB0145480 | C16H13NO6 | 0.623 | 0.910 | -0.136 | 0.189 |  | 0.722 | 0.828 | -0.272 | 0.006 |
| 30 | ESI- | 321.1008 | 1.15 | D-Pantothenoyl-L-cysteine | HMDB0006834 | C12H22N2O6S | 0.580 | 1.330 | 0.412 | 0.283 |  | 0.723 | 1.875 | 0.907 | 0.005 |
| 31 | ESI- | 348.1 | 1.17 | S-(Formylmethyl)glutathione | HMDB0060507 | C12H19N3O7S | 0.514 | 1.121 | 0.164 | 0.617 |  | 0.724 | 2.024 | 1.017 | 0.004 |
| 32 | ESI- | 149.0444 | 1.1 | D-Xylulose | HMDB0001644 | C5H10O5 | 0.558 | 0.973 | -0.040 | 0.824 |  | 0.726 | 1.905 | 0.929 | 0.002 |
| 33 | ESI+ | 263.1384 | 4.81 | Prolylphenylalanine | HMDB0011179 | C14H18N2O3 | 0.582 | 0.964 | -0.053 | 0.829 |  | 0.740 | 0.711 | -0.493 | 0.011 |
| 34 | ESI- | 87.0454 | 1.2 | Butyric acid | HMDB0000039 | C4H8O2 | 0.652 | 1.593 | 0.672 | 0.056 |  | 0.743 | 3.156 | 1.658 | 0.014 |
| 35 | ESI- | 236.0916 | 1.26 | 1-Carboxyethylphenylalanine | HMDB0240628 | C12H15NO4 | 0.531 | 1.178 | 0.236 | 0.476 |  | 0.734 | 1.724 | 0.785 | 0.008 |
| 36 | ESI- | 144.0668 | 1.12 | (S)-2-amino-6-oxohexanoate | HMDB0001263 | C6H11NO3 | 0.569 | 1.273 | 0.349 | 0.181 |  | 0.736 | 2.101 | 1.071 | 0.003 |
| 37 | ESI- | 297.1188 | 1.11 | LysoPA(8:0/0:0) | HMDB0114740 | C11H23O7P | 0.569 | 1.328 | 0.409 | 0.239 |  | 0.730 | 2.485 | 1.313 | 0.012 |
| 38 | ESI+ | 333.1452 | 7.25 | Glutaminyltryptophan | HMDB0028808 | C16H20N4O4 | 0.673 | 2.274 | 1.185 | 0.164 |  | 0.779 | 2.132 | 1.092 | 0.050 |
| 39 | ESI- | 389.0879 | 3.6 | Dopaxanthin | HMDB0012221 | C18H18N2O8 | 0.624 | 11.098 | 3.472 | 0.110 |  | 0.790 | 4.418 | 2.143 | 0.035 |
| 40 | ESI- | 181.9656 | 1.06 | Se-Methylselenocysteine | HMDB0004113 | C4H9NO2Se | 0.691 | 0.712 | -0.491 | 0.077 |  | 0.791 | 0.523 | -0.935 | 0.000 |
| 41 | ESI- | 239.0162 | 0.97 | L-Cystine | HMDB0000192 | C6H12N2O4S2 | 0.545 | 1.324 | 0.405 | 0.112 |  | 0.791 | 1.610 | 0.687 | 0.000 |
| 42 | ESI+ | 315.2535 | 20.8 | 12,13-DHOME | HMDB0004705 | C18H34O4 | 0.584 | 0.955 | -0.066 | 0.902 |  | 0.783 | 0.337 | -1.571 | 0.001 |
| 43 | ESI- | 96.9611 | 0.9 | Sulfate | HMDB0001448 | H2O4S | 0.508 | 1.062 | 0.086 | 0.708 |  | 0.780 | 1.645 | 0.718 | 0.001 |
| 44 | ESI+ | 257.0745 | 1.22 | 7-hydroxy-8-(sulfooxy)  octanoic acid | HMDB0159457 | C8H16O7S | 0.645 | 0.891 | -0.167 | 0.110 |  | 0.827 | 0.685 | -0.546 | 0.000 |
| 45 | ESI+ | 403.3571 | 20.9 | 7alpha-Hydroxycholesterol | HMDB0001496 | C27H46O2 | 0.559 | 1.158 | 0.211 | 0.699 |  | 0.823 | 0.345 | -1.536 | 0.000 |
| 46 | ESI+ | 112.8955 | 1.03 | Selenium Sulfide | HMDB0015106 | SSe | 0.670 | 0.872 | -0.197 | 0.064 |  | 0.809 | 0.766 | -0.385 | 0.000 |
| 47 | ESI- | 415.2058 | 4.72 | Cyclic Phosphatidic acid(18:2) | HMDB0007007 | C21H37O6P | 0.688 | 2.668 | 1.416 | 0.052 |  | 0.803 | 3.876 | 1.955 | 0.001 |

**Table S3 Metabolites whose difference may affect the clinical phenotypes on cirrhosis and decompensation.**

| **Metabolite** | **Description** | **Increased or decreased in cirrhosis** | **Exist in cirrhosis-associated fingerprint** |
| --- | --- | --- | --- |
| **Related to lipids** |  |  |  |
| Docosadienoyl-CoA | Docosadienoyl-CoA, belongs to the class of organic compounds known as very long-chain fatty acids. | ↓ | ◊ |
| DG(15:0/20:5(5Z,8Z,11Z,14Z,17Z)/0:0) | It is a diacylglycerol (DAG).Synthesis of diacylglycerol begins with glycerol-3-phosphate, which is derived primarily from dihydroxyacetone phosphate, a product of glycolysis (usually in the cytoplasm of liver or adipose tissue cells). | ↓ | † |
| Ganglioside GM3 (d18:1/12:0) | Ganglioside GM3 (d18:1/12:0) is a component the cell plasma membrane which modulates cell signal transduction events. Gangliosides have been found to be highly important in immunology. | ↓ | ◊ |
| trans-2-Enoyl-OPC4-CoA | Trans-2-enoyl-OPC4-CoA belongs to the class of organic compounds known as 2-enoyl CoAs, participating in alpha-Linolenic acid metabolism. | ↓ | ◊ |
| Tetracosatetraenoyl CoA | Tetracosatetraenoyl CoA, also known as etracosatetraenoic acid, belongs to the class of organic compounds known as very long-chain fatty acyl coas. | ↓ | ◊ |
| (S)-3-Hydroxydodecanoyl-CoA | It is also known as (S)-3-hydroxylauroyl-CoA, belongs to the class of organic compounds known as (s)-3-hydroxyacyl coas. | ↓ | ◊ |
| TG(16:1(9Z)/18:0/20:4(5Z,8Z,11Z,14Z)) | TGs are major components of very low density lipoprotein (VLDL) and chylomicrons, play an important role in metabolism as energy sources and transporters of dietary fat.Fat cells can synthesize and store triglycerides. | ↓ | ◊ |
| (2E)-Decenoyl-CoA | (2E)-Decenoyl-CoA is a beta-oxidation intermediate, the substrate of the enzyme peroxisomal acyl-CoA thioesterase 2 (PTE-2, 3.1.2.2), which is localized in the peroxisome. | ↓ | ◊ |
| Galabiosylceramide (d18:1/26:0) | Galabiosylceramide is a non-acidic diglycosphingolipids, i.e. a sphingolipid with two or more carbohydrate moieties attached to a ceramide unit. It is a vital component of cellular membranes of most eukaryotic organisms and some bacteria. | ↓ | ◊ |
| Cyclic Phosphatidic acid(18:2) | CPA(18:2) is a cyclic phosphatidic acid or cyclic lysophosphatidic acid.In particular, it is found in tissues subject to injury, and while it may have some similar signalling functions to lysophosphatidic acid per se, it also has some quite distinct biological activities. For example, cyclic phosphatidic acid is known to be a specific inhibitor of DNA polymerase alpha. | ↑ | † |
| N-tetradecanoylsphinganine | N-tetradecanoylsphinganine is a ceramide (Cer),Ceramide phosphates are important metabolites of ceramides as they act as a mediators of the inflammatory response.Ceramides can also be synthesized from serine and palmitate in a de novo pathway and are regarded as important cellular signals for inducing apoptosis. | ↓ |  |
| 12,13-DHOME | 12,13-DHOME is a derivative of the linoleic acid diol that has been reported to be toxic in human tissue preparations. It is a naturally occurring proliferator-activated receptor (PPAR) gamma2 ligand, which stimulates adipocytes and inhibits osteoblast differentiation | ↓ | ◊ |
| PE(24:1(15Z)/P-18:0) | PE(24:1(15Z)/P-18:0) is a phosphatidylethanolamine (PE or GPEtn). It is a glycerophospholipid in which a phosphorylethanolamine moiety occupies a glycerol substitution site. | ↓ |  |
| DG(15:0/20:5(5Z,8Z,11Z,14Z,17Z)/0:0) | It is a diglyceride, or a diacylglycerol (DAG).Synthesis of diacylglycerol begins with glycerol-3-phosphate, which is derived primarily from dihydroxyacetone phosphate, a product of glycolysis (usually in the cytoplasm of liver or adipose tissue cells). | ↓ |  |
| Sphinganine 1-phosphate | Sphingosine-1-phosphate is a signaling sphingolipid, also known as lysosphingolipid. It is also referred to as a bioactive lipid mediator. | ↑ | ◊ |
| Trihexosylceramide (d18:1/26:1(17Z)) | Trihexosylceramide is a glycosphingolipid which contains a trisaccharide (galactose-galactose-glucose) moiety bound in glycosidic linkage to the hydroxyl group of ceramide as the polar head group. | ↓ | ◊ |
| LysoPA(8:0/0:0) | LysoPA(8:0/0:0) is a lysophosphatidic acid.Lysophosphatidic acid is the simplest possible glycerophospholipid. It is the biosynthetic precursor of phosphatidic acid. Although it is present at very low levels only in animal tissues, it is extremely important biologically, influencing many biochemical processes. | ↑ |  |
| **Related to bile acids and bile secretion** |  |  |  |
| 2-Hydroxyethanesulfonate | 2-Hydroxyethanesulfonate (also known as 2-Hydroxyethanesulfonic acid or isethionic acid) is an organosulfur compound containing a short chain alkane sulfonate linked to a hydroxyl group. Higher plasma levels of 2-hydroxyethanesulfonate have been shown to be protective against type 2 diabetes. | ↑ | ◊ |
| Taurodeoxycholic acid | Taurodeoxycholic acid is a bile salt formed in the liver by conjugation of deoxycholate with taurine, usually as the sodium salt. Bile acids are steroid acids found predominantly in the bile of mammals. | ↑ | ◊ |
| 3α,7α-Dihydroxy-5β-cholestane | 3α,7α-Dihydroxy-5β-cholestane is an intermediate in bile acid synthesis. Bile acids are steroid acids found predominantly in the bile of mammals. Bile acids are physiological detergents that facilitate excretion, absorption, and transport of fats and sterols in the intestine and liver. | ↓ |  |
| Sulfolithocholylglycine | Sulfolithocholylglycine is an acyl glycine and a bile acid-glycine conjugate. It is a secondary bile acid produced by the action of enzymes existing in the microbial flora of the colonic environment.Sulfolithocholylglycine is a sulfated bile acid which has a greater renal clearance rate than lithocholylglycine. | ↓ |  |
| 7α-Hydroxycholesterol | 7α-Hydroxycholesterol is an oxysterol and can serve as a biomarker for lipid peroxidation. Products of cholesterol oxidation accumulate within atherosclerotic plaque and have been proposed to contribute to inflammatory signalling in the diseased artery. | ↓ |  |
| **Related to amino acids and proteolysis** |  |  |  |
| L-Kynurenine | Kynurenine is a metabolite of the amino acid tryptophan used in the production of niacin. L-Kynurenine is a central compound of the tryptophan metabolism pathway since it can change into the neuroprotective agent kynurenic acid or to the neurotoxic agent quinolinic acid. | ↑ | ◊ |
| Threonyl-prolyl-arginyl-lysine | Threonyl-prolyl-arginyl-lysine (also called Kentsin) is an incomplete breakdown product of protein catabolism. | ↑ | ◊ |
| Se-Methylselenocysteine | Se-Methylselenocysteine (SeMSC) is a naturally occurring seleno-amino acid.SeMSC is not incorporated into any proteins, thereby being fully available for the synthesis of selenium-containing enzymes such as glutathione peroxidase. | ↓ | † |
| Hydroxyprolyl-Tyrosine | Hydroxyprolyl-Tyrosine is a dipeptide composed of hydroxyproline and tyrosine. It is an incomplete breakdown product of protein digestion or protein catabolism. | ↑ |  |
| Histamine | Histamine is an organic nitrogenous compound involved in local immune responses, as well as regulating physiological function in the gut and acting as a neurotransmitter for the brain, spinal cord, and uterus. | ↓ |  |
| Prolylphenylalanine | Prolylphenylalanine is a dipeptide composed of proline and phenylalanine. It is an incomplete breakdown product of protein digestion or protein catabolism. | ↓ |  |
| Glutaminyltryptophan | Glutaminyltryptophan also known as Q-W dipeptide or L-GLN-L-TRP, belongs to the class of organic compounds known as dipeptides. | ↑ |  |
| L-Cystathionine | L-Cystathionine is a modified amino acid generated by enzymic means from L-homocysteine and L-serine. | ↑ |  |
| Glutaminylphenylalanine | Glutaminylphenylalanine, also known as Q-F dipeptide or L-GLN-L-phe, belongs to the class of organic compounds known as dipeptides. | ↑ |  |
| D-Pantothenoyl-L-cysteine | D-Pantothenoyl-L-cysteine is involved in the pantothenate and CoA biosynthesis pathway. | ↑ |  |
| S-(Formylmethyl)glutathione | S-(Formylmethyl)glutathione is a S-substituted glutathione. This compound belongs to the family of Peptides | ↑ |  |
| 1-Carboxyethylphenylalanine | 1-Carboxyethylphenylalanine belongs to the class of organic compounds known as phenylalanine and derivatives. | ↑ |  |
| (S)-2-amino-6-oxohexanoate | (S)-2-amino-6-oxohexanoate also known as Allysine.Allysine is a derivative of lysine used in the production of elastin and collagen and is essential in the crosslink formation that stabilizes collagen and elastin. | ↑ |  |
| L-Cystine | L-Cystine functions as an antioxidant and protects tissues against radiation and pollution, slowing the aging process, also aids protein synthesis. | ↑ |  |
| N-Acetylglutamine | N-Acetylglutamine is used for parenteral nutrition as a source of glutamine, since glutamine is too unstable, but N-Acetylglutamine is very stable. | ↓ |  |
| **Related nicotinate and nicotinamide** |  |  |  |
| 1-(beta-D-Ribofuranosyl)-1,4-dihydronicotinamide | 1-(beta-D-Ribofuranosyl)-1,4-dihydronicotinamide is the reduced form of nicotinamide riboside. | ↓ | † |
| N1-Methyl-4-pyridone-3-carboxamide | In humans, N1-methyl-4-pyridone-3-carboxamide is involved in nicotinate and nicotinamide metabolism. | ↑ | † |
| 6-Hydroxynicotinic acid | 6-hydroxynicotinic acid is a monohydroxypyridine that is the 6-hydroxy derivative of nicotinic acid. | ↓ | ◊ |
| **Related to nucleoside** |  |  |  |
| Mesobilirubinogen | Mesobilirubinogen(Urobilinogen) is an uribiniloid, the product of bilirubin reduction in multiple sequential reactions. | ↑ | ◊ |
| Guanosine 3',5'-bis(diphosphate) | Guanosine 3',5'-bis(diphosphate), also known as guanosine-5',3'-tetraphosphATE or tetraphosphate, guanosine, belongs to the class of organic compounds known as phenylpyruvic acid derivatives.This nucleotide serves as a messenger to turn off the synthesis of ribosomal RNA when amino acids are not available for protein synthesis. | ↓ | ◊ |
| Bis(5'-guanosyl) tetraphosphate | In humans, diguanosine tetraphosphate is involved in the metabolic disorder called the purine nucleoside phosphorylase deficiency pathway. | ↓ | ◊ |
| Glycineamideribotide | Within humans, glycineamideribotide participates in a number of enzymatic reactions. | ↓ |  |
| Pseudouridine | C-glycoside isomer of the nucleoside uridine; involved in translation of mRNA into proteins; the most prevalent of the over one hundred different modified nucleosides found in RNA; enhances the function of transfer RNA and ribosomal RNA by stabilizing the RNA structure. | ↑ |  |
| **Related to glucoside and carbohydrates** |  |  |  |
| 6-Phosphogluconic acid | Within humans, 6-phosphogluconic acid participates in a number of enzymatic reactions. In particular, 6-phosphogluconic acid can be biosynthesized from gluconolactone; which is mediated by the enzyme 6-phosphogluconolactonase. | ↓ | † |
| D-Xylulose | D-xylulose is converted from xylitol by the enzyme NAD+-linked xylitol dehydrogenase in the glucuronate pathway, the most important xylitol-handling metabolic pathway in mammals. | ­ |  |
| **Related to intermediate metabolites** |  |  |  |
| ADP | It is an ester of pyrophosphoric acid with the nucleotide adenine. ADP consists of the pyrophosphate group, the pentose sugar ribose, and the nucleobase adenine. ADP is the product of ATP dephosphorylation by ATPases. ADP is converted back to ATP by ATP synthases. | ↓ | ◊ |
| 2-Piperidinone | It has a role as an L-glutamate gamma-semialdehyde dehydrogenase inhibitor. | ­ |  |
| Harderoporphyrinogen | Harderoporphyrinogen belongs to the class of organic compounds known as porphyrins. | ­ | ◊ |
| 3-Methoxyanthranilate | It is a metabolite of kynurenine. | ­ | ◊ |
| Trimethylamine | Trimethylamine has been found in human muscle and epidermis tissues. | ­ | ◊ |
| Phenylacetaldehyde | Phenylacetaldehyde is one important oxidation-related aldehyde. | ­ |  |
| **Related to other acids** |  |  |  |
| 2-Oxo-4-methylthiobutanoic acid | It is a member of the class of compounds known as thia- fatty acids. | ↓ | ◊ |
| D-Phenyllactic acid | Phenylacetaldehyde is one important oxidation-related aldehyde. | ­ | ◊ |
| 7-hydroxy-8-(sulfooxy)octanoic acid | It is considered to be a fatty acid lipid molecule. | ↓ |  |
| Butyric acid | Butyric Acid is a saturated short-chain fatty acid with a 4-carbon backbone. | ­ | † |
| Traumatic acid | Traumatic Acid can cause a decrease in membrane phospholipid peroxidation and show antioxidant and stimulatory effects on collagen biosynthesis. | ↓ | ◊ |
| 2-methylbut-3-enoic acid | It is the class of organic compounds known as branched fatty acids. | ↑ | ◊ |
| **Related to bacteria metabolism** |  |  |  |
| dTDP-4-dehydro-6-deoxy-α-D-glucose | A dTDP-sugar having 4-dehydro-6-deoxy-α-D-glucose as the sugar component. It is an intermediate in dTDP-rhamnose biosynthesis. | ↓ | ◊ |
| Pimelic acid | Pimelic acid, also known as heptanedioic acid or heptanedioate, belongs to the class of organic compounds known as medium-chain fatty acids. | ↓ | ◊ |
| Paromomycin | Paromomycin produced by Streptomyces riomosus var | ↑ | ◊ |
| 2-(hydroxylamino)benzoic acid | It is a bacterial metabolite. It is an aminobenzoic acid and a member of hydroxylamines. | ↑ | ◊ |
| 3-hydroxy-4-methoxy-5-(3,4,5-trihydroxybenzoyloxy)benzoic acid | It is a predicted metabolite generated by BioTransformer¹ that is produced by the metabolism of 3,4-dihydroxy-5-(3,4,5-trihydroxybenzoyloxy)benzoic acid. | ↓ | ◊ |
| 2,4,6-trihydroxybenzaldehyde | 2, 4, 6-trihydroxybenzaldehyde is a predicted metabolite generated by BioTransformer. | ↑ |  |
| **Uncategorized** |  |  |  |
| [4-(3,7-dihydroxy-4-oxo-3,4-dihydro-2H-1-benzopyran-2-yl)phenyl]oxidanesulfonic acid | It related to oxidanesulfonic acid. | ↓ | ◊ |
| 3,4,5-trihydroxy-6-{[(4Z,10Z,12E,14E,19Z)-7,16,17-trihydroxydocosa-4,8,10,12,14,19-hexaenoyl]oxy}oxane-2-carboxylic acid | It related to carboxylic acid. | ↓ | ◊ |
| xi-2,3-Octadiene-5,7-diyn-1-ol | It belongs to the class of organic compounds known as fatty alcohols. | ↑ | † |
| Dopaxanthin | Dopaxanthin quinone is a non-proteinogenic alpha-amino acid. | ↑ | † |
| Inosine | Inosine is commonly found in tRNAs and is essential for proper translation of the genetic code in wobble base pairs. | ↑ | ◊ |
| 2,2-dichloroethane-1,1-diol | It is a intermediary metabolite of dichloroethane. | ↓ |  |
| 3-Methoxy-4-hydroxyphenylethyleneglycol | It is a metabolite of phenylalanine. | ↓ |  |
| 3-methoxyphenol | It is also known as 3-hydroxyanisole or m-guaiacol, belongs to the class of organic compounds known as methoxyphenols. | ↑ |  |
| 2-({hydroxy[2-(2-hydroxybenzoyloxy)phenyl]methylidene}amino)acetic acid | | ↓ |  |
| Selenium Sulfide | It is the form of the element Selenium. | ↓ |  |
| Biotin | Biotin is used to prevent or treat low levels of vitamin B7 in people who do not get enough of the vitamin from their diets. | ↑ |  |
| 3-Hydroxy-3-methylcyclohexan-1-one | It is a intermediary metabolite. | ↑ |  |
| (3,4-dihydroxyphenyl)methyl 7-methyloctanoate | It is a intermediary metabolite. | ↓ |  |
| {[(8E)-10,15-dioxoheptadeca-8,16-dien-11,13-diyn-7-yl]oxy}sulfonic acid | It is a metabolite of sulfonic. | ↑ |  |
| Sulfate | Sulfates, compounds composed of sulfate ions (SO42-) and other metal ions, are electrolytes | ↑ |  |
| ◊There are cirrhosis-associated fingerprints; † There are fingerprints associated with both cirrhosis and decompensation. | | | |
